# The Role of Housing Tenure Opportunities in the Social Integration of the Aging Pre-1970 Migrants in Beijing

**DOI:** 10.3390/ijerph19127093

**Published:** 2022-06-09

**Authors:** Ye Zhu, Weiyu Cao, Xin Li, Ran Liu

**Affiliations:** College of Resource Environment and Tourism, Capital Normal University, No. 105 West 3rd Ring Road North, Beijing 100048, China; 1190902032@cnu.edu.cn (Y.Z.); 1190902002@cnu.edu.cn (W.C.); 1190902010@cnu.edu.cn (X.L.)

**Keywords:** aging migration, housing tenure, social integration, Beijing

## Abstract

This study focuses on the social integration of the pre-1970 first-generation migrants in reformist China, who were born before the year 1970 while getting old in the destination cities. The pre-1970 first-generation migrants are not a homogeneous group but are composed of: (a) those over 45 years old and still working but facing age discrimination; and (b) the elderly granny as nanny assuming the domestic and child-care work for their sons or daughters in the destination cities. We conceptualized and re-defined the aging migrants’ social integration into three dimensions (i.e., participation practices, communication contacts, and subjective perceptions), and used the 2017 Migrant Dynamics Monitoring Survey (MDMS) data from Beijing to measure and explain the varied integration levels among a total of 1267 aging migrant samples in the Beijing metropolis. It is proven that housing tenure matters and housing tenure entitlement would be conductive to beefing up aging migrants’ integration. However, informal housing should not be “stigmatized” as a segregated world, since those dwelling in the informal housing have reported a higher probability of perceiving a fully integrated status (namely subjective well-being, SWB) than those living in the dormitory-like housing. Additionally, an employment-income paradox is found, which shows that higher economic achievement is NOT equivalent to a higher social integration status for the aging migrants.

## 1. Introduction

The integration and health of the aging migrants have become a global issue in recent years and attracted much attention in both the OECD countries and the developing world [1,2]. The aging migrants are easier exposed to integration and health risks owing to their floating and therefore disadvantaged socioeconomic status, and a series of cultural, institutional, and language barriers have associations with their social integration and health-seeking behaviors. Existing studies have revealed such disadvantages, vulnerability, and discrimination facing the aging migrants in different contexts. The healthy aging schemes, however, have not expanded the health and quality of life-related benefit packages to cover the entire spectrum of migrant populations (especially aging migrants) in many countries. For instance, the World Health Organization (WHO) and its members had created a Global Strategy and Action Plan for Ageing and Health (2016–2020), stressing that everyone can live a long and healthy life [3]. However, this Global Strategy mentioned little about the disadvantageous aging migrants and their integration and health benefits. In recent years, the European Healthy Cities Network began to target the aging migrants (for instance, migrant women of Moroccan origin aged 55 years and over in the specific context of Rotterdam) at risk of loneliness [4]. Other case studies from Italy, Finland, Sweden, and Ireland of the European Healthy Cities Network had proposed a whole-of-society approach to target the vulnerable groups and their related networks (e.g., families and migrant communities) to catalyze their integration across society [4].

Similar to the process of population aging and increased migration that have close associations with the demographics of Europe, the aging migrants have been an important part of China’s urban population, too. Massive migration in China began after market-oriented reforms at the end of the 1970s when China relaxed its *hukou*-based household registration system and allowed its populations to move freely between different cities or from rural to urban areas. This great transformation marked a historical turning point in the 1970s. The pre-1970 generation (who were born before 1970) has been regarded as the first-generation migrants in reformist China. Migrants who were born during the 1980s, 1990s, and 2000s are termed the “New Generation Migrants” in China, namely the post-1980, post-1990, and post-2000 generations [5]. The existing studies noticed a sea-change in the industrial and labor relations and societal structures faced by the new generation of migrants [6]. Little attention, however, was given to the social integration of the “aging first-generation migrants” (the pre-1970 generation born before the year 1970) in China.

The pre-1970 migrants have a wide range of age cohorts in urban China: (a) those over 45 years old and still working; and (b) de facto elderly migrants already retired. Such an aging process deserves attention, as it would incur more risk factors for exclusion and disease. For instance, some recent research began to stress the unemployment and exclusion risks exposed to people over 45 years old (as a specific vulnerable group in the employment market) in times of crisis and restructuring in both the OECD and developing countries. A study on Spanish unemployment revealed the socio-professional context that rejects and excludes people over 45 years old from the job market [7]. The empirical studies in the UK confirmed this conclusion [8]. Another research on the age limit in recruitment advertisements in China also pointed out the age discrimination against job seekers: the upper age limit of selected four types of jobs (handyman, technician, engineer, and manager) has a trend of convergence towards 45 years old [9]. The de facto elderly migrants who are already retired also face difficulties adapting to the living environments, because they assume the domestic and child-care work (namely granny as a nanny) for their sons or daughters but have few chances for out-of-home leisure or local communication contact. The above continuity but heterogeneity of the aging process (ranging from the migrant labor over 45 years to the retired granny) deserves further investigation.

In this paper, we tentatively re-define the social integration dimensions of the aging pre-1970 migrants in Beijing. The aging- and migrant-specific integration-seeking behaviors and perceptions matter, involving (a) aging migrants’ participation in local affairs (rather than engagement with their own clan/lineage), (b) extensiveness of communication contacts (migrant-local ties, rather than within migrant networks), and (c) self-identification and perceptions of their social integration status. The specific measures of participation, communication contact, and perception are clarified. We then outline the key demographic, migratory, and contextual factors that shape the differentiated social integration profiles of the aging migrants in the transitional Beijing metropolis. Recent studies have proved that better housing opportunities are accompanied by a higher level of social integration [10]. However, relevant studies focused only on the physical context (e.g., physical quality, characteristics, arrangement, and environment) of housing [11], and did not mention the housing tenure context. We innovatively list the housing tenure (as an outcome of migrants’ self-selection and socio-spatial stratification) as the contextual factor, which has associations with the social interaction behaviors and perceptions of the aging pre-1970 migrants. This research uses the Migrant Dynamics Monitoring Survey (MDMS) data in 2017 in the Beijing metropolis and employs logistic regression analysis to attest to whether the housing opportunities (owning, renting, employer- or government-provided, informality) have significant associations with the social integration of the aging pre-1970 migrants in Beijing. In conclusion, there is a need for more attention given to the healthy aging scheme providing the aging migrants better housing opportunities, creating a positive sense of self, and more chances for participation, communication, and empowerment. This is important to accommodate the needs of the growing number of aging migrants in China.

## 2. Social Integration of Aging Migrants in China: Review and Conceptualization

### 2.1. Migration, Aging, and the Role of the Housing Regime in the Social Stratification and Inclusion

In contrast to the Western countries, the household registration system (namely *hukou*) is a particular institution that links people’s place of origin to their daily life, social services, and welfare in China, and it has exerted an impact on the social stratification between local vs. nonlocal *hukou* holders and between talented vs. less-skilled migrant workers [12,13]. The New-Styled Urbanization Plan and *hukou* reforms have been implemented during the 13th Five-Year Plan period (2015–2020) to integrate migrants in terms of employment opportunities, housing acquisition, and social services delivery [14]. The recent *hukou* reform ensured that the migrants can get urban *hukou* in medium-sized cities (with three million residents and below), but *hukou* restrictions remain strong in first-tier cities and megacities such as Beijing. Megacities began to open their *hukou* doors to talented youth, although policy makers still stress the importance of the *hukou* system to avoid urban ills and population over-crowding [15].

Migrants’ integration and willingness to settle in the destination cities have become the focus of current research on migration in China. The existing studies revealed the positive impact of human capital and social networks on the migrants’ socioeconomic and psychological integration [16,17]. More recent research began to compare migrants’ integration in the different neighborhood types in urban China [18,19,20] since neighbor networks and socioeconomic and physical environments of neighborhoods are vital components of integration [21,22,23].

China has the largest elderly population in the world and its pace of aging (i.e., fast-growing number and proportion of the elderly people in cities) has become rapid in recent years. However, relatively little attention has been given to the pre-1970 generation migrants (born before 1970), who were the first-generation migrants in reformist China who are aging now. In recent years, researchers began to discuss the pre-1970 migrants’ social integration issues. For instance, Lin and Huang used the 2015 national scale data issued by the China Household Financial Survey (CHFS) to analyze the cohort differences among migrants, concerning the community environmental satisfaction and their impact on migrants’ subjective well-being (SWB) [24]. It was found that the satisfying social environment (rather than the physical environment) exhibited the positive associations with happiness among the pre-1970 migrants, and at the same time, the positive associations of a satisfying social life with SWB were more observable in the pre-1970 migrants than their younger cohorts. It was found that the social integration (including social environment and social life as revealed in Lin and Huang’s studies) exerts a prominent impact on the health and quality of life of the pre-1970 first-generation migrants. However, little is known regarding the “localized” participation and communication behaviors and perceptions of social integration among the pre-1970 migrants, who are getting old.

The pre-1970 migrants straddle a wide range of life courses. They are facing quite different situations under China’s retirement policy, if they are under 60 years old (mostly still working) or over 60 years old (mostly retired). More attention needs to be put on the sizeable aging migrants in China, including (a) de facto elderly migrants (mostly retired, living with their sons or daughters and caring for grand-children in the destination city), and (b) aging-but-still working migrant workers (still employed but relatively disadvantaged compared with the post-1980 and post-1990 new generations in terms of their adaptation to the new economies in megacities). The aging migrants are called “*laopiao*”, having left the familiar environment and social networks in their hometown and floating in a destination city, either as aging migrant workers or granny as a nanny. Such aging migration in transitional China is not for recreation or retirement purposes but is an economic or family-unification driven migration. The aging migrants face difficulties adapting to the living environments in megacities (such as Beijing), regardless of whether co-residing with their children or not. If they assume the domestic and child-care work for their sons or daughters, they have fewer chances for out-of-home leisure and local communication contacts [25]. As revealed, the language barrier can reduce the elderly migrants’ abilities to build up a social network in the destination city and result in poorer health conditions [26]. The older migrants face mobile vulnerability in big cities such as Beijing, but they can develop coping strategies to improve their socio-cultural mobility in the destination [27]. It is also found that social environment and social activities can contribute to old adults’ health in the megacities such as Shanghai [28]. Scholars have examined the impact of different neighborhood types on the quality of life among elderly people in Nanjing and Beijing megacities [29,30]. These aging and healthy living studies, however, have not been extended to the aging migrants who are more disadvantaged than the local elderly people.

Less attention has been paid to the role of housing types in integration. Housing provides housing security, public services, and upward mobility opportunities (such as school provisions organized around the catchment areas) for the migrants [31]. In China, *hukou* imposes limits on the social integration of migrants, but recent studies have indicated that the migrants tend to achieve permanent settlement through more flexible channels such as purchasing urban housing in the destination city, even though the migrants have limited housing opportunities than residents since that house prices are particularly high in big cities [32,33]. Moreover, housing is a symbol of the living quality of the migrants and an important indicator and a result of social stratification in the destination city in China [34,35]. As early as the 1960s, the new Weber School represented by Rex put forward the concept of housing class and emphasized that individual possession of housing tenure is one of the criteria for dividing the social classes [36]. Housing type or housing choice is the primary factor directly related to the migrants’ social stratification and inclusion, especially in transitional economies such as China with a profound housing commodification reform since the 1990s. Interestingly, the concept of “housing type” and “community type” is seemingly exchangeable in the previous studies on the so-called community-level integration studies. For instance, it is proved that migrants living in the government-sponsored “affordable housing” community exhibited a much higher level of social integration than those living in other “rental” communities [37]. It is also found that migrants who live in “commodity housing” neighborhoods achieve a higher level of integration, but those living in “urban villages” cannot find a path to integrate into the mainstream city [20,38,39]. What is more important, housing tenure itself would stimulate interaction and integration [40]. The recent studies on neighborhood-level integration also stressed the significance of housing tenure type. A recent empirical study on migrants in peri-urban neighborhoods in Beijing pointed out that a low sense of belonging among migrants is not necessarily a result of homogenous tenure, but of living with uncertainty and exclusion from the formal urban economy, and scholars have revealed the role of tenure heterogeneity in providing a sense of privilege and privacy [19]. It is uncertain whether this conclusion can be held in other contexts, but housing type indeed matters in the integration process. In addition, housing unaffordability is found negatively correlated with migrants’ mental health, and compared with migrants who are home-occupiers, the tenant migrants are more easily excluded from the psychological integration in the face of high rental costs [41,42]. However, we know little about the role of housing stratification in inclusion/exclusion processes, especially among the aging migrant populations.

### 2.2. Conceptualization, Framework, and Hypotheses

The existing studies on the migrants’ social integration under China’s *hukou* regime have measured the willingness to settle down or not, and the willingness to make *hukou* conversion or not, as a representation of the migrants’ sense of belonging and identity to the city [43,44]. However, for older migrants, having or lacking strong ties/networks with locals, and engaging in local affairs are profoundly important predictors of well-being and health status. As pointed out by the Sociologist Louis Wirth, “If men of diverse experiences and interests are to have ideas and ideals in common, they must have the ability to communicate”. The integration process requires the extensiveness of contact among migrant populations and local society [45,46]. Existing studies, however, tended to define integration to be a small network within the neighborhoods. The social integration interpretation (as well as integration into local society in general) should build up and maintain the larger networks (such as the city-wide housing tenure system, the urban-bound participation activities, and perceptions) rather than neighborhood-bound within some specific mosaic-like enclaves or the recommended socio-ethnically mixed neighborhoods. The previous studies had tried to prove that the overall best direct predictor of social integration was community or neighborhood [47,48]. The Western experience focused on the associations of the mixed neighborhood with integration and believed that physical mixing can promote interclass interaction [49]. However, Musterd, Ostendorf, and Galster pointed out it is unclear whether a neighborhood mixing between different people does indeed result in a higher level of integration [50,51]. The community-level mechanism of integration is still opaque, and the analysis results vary with the specific contexts.

Ager and Strang proposed a conceptual framework for understanding integration in 2008, and they listed the key domains of integration: (a) achievement and access across the sectors of employment, housing, education, and health; (b) practices regarding citizenship and rights; (c) processes of social connection within and between groups; and (d) structural barriers to such connection related to language, culture and the local environments [52]. Among them, housing is stressed as one of the most researched areas of integration, and as important as employment and education. They paid attention to the associations that housing has with the physical and emotional well-being, the financial security of tenancies and ownership, their ability to feel at home, settled (as well as the continuity of relationships associated with being settled), safe, secure and stable, and other social and cultural impact of housing. Ager and Strang’s conceptualizations are likely to remain controversial [53], but they can embrace the significant questions regarding the specific processes that can facilitate integration (for instance, concerning targeted housing policies that can make opportunities for social connection or a sense of safety) [52]. Chen and Wang adopted Ager and Strang’s conceptual framework on what is integration and developed the three-dimensional measurement of the Chinese new generation migrants’ social integration process: (a) participation in community activities; (b) connection with the community, and (c) accustomed to social norms [54]. They then used the relevant factors and processes that facilitate integration to explain the younger migrants’ integration in Shanghai.

It is important to see the aging migrants’ integration as a practical and relational process and multiple measurements, not a purely perceptual outcome concerning satisfaction, happiness, or willingness to settle down or make *hukou* conversion to a destination city. Here, as elaborated by Robert Brown (2010: 2), the social integration refers to the range of a person’s communication contacts (regardless of individual-level, small group, or mass communication) rather than to their frequency of communication, as the integration process requires some extensiveness of communication contacts [46]. We can assume that integration is itself dependent on extensive communication with the local population and that migrants who report a variety of communication contacts with the locals differ from those who report few or no such contacts.

Because housing choice and housing type are vital components of social stratification statuses and city/neighbor networks, we examine the housing stratification-related integration perspectives to explain the impact of “housing tenure type” on the aging pre-1970 migrants’ “integration” in the Chinese metropolises such as Beijing. The aging migrants’ social integration is defined from three dimensions (i.e., participation practices, communication contacts, and subjective perceptions, similar to Ager and Strang’s conceptualization and Chen and Wang’s adoption of it in the Chinese megacity contexts [52,54]):

(a) Their actual participation in local affairs (regardless of a small group-like community/neighborhood or the mass society of a city);
(b) Communication contacts with the locals, beyond their own patriarchal family- and clan-relationship (namely “*laoxiang*” as people from similar originating areas); and
(c) Migrants’ relevant perceptions of subjective well-being (SWB).

Can a housing tenure regime be better designed to promote the aging migrants’ social integration with their local social capital and environments? Next, we will use the survey evidence from the 2017 Migrant Dynamics Monitoring Survey (MDMS) data in Beijing and the logistic regression to examine this possibility by testing the relationships between varying types of housing tenure and the strength of integration: (a) whether housing tenure opportunities can predict the aging migrants’ social integration; and (b) which kind of housing tenure attainment/arrangement is more conducive to (or can best support) the integration of aging migrants in the metropolises such as Beijing?

**Figure 1:**
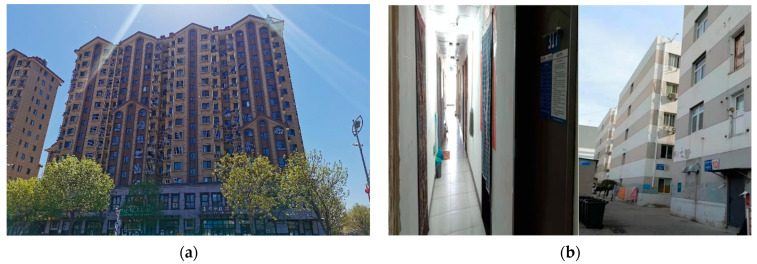

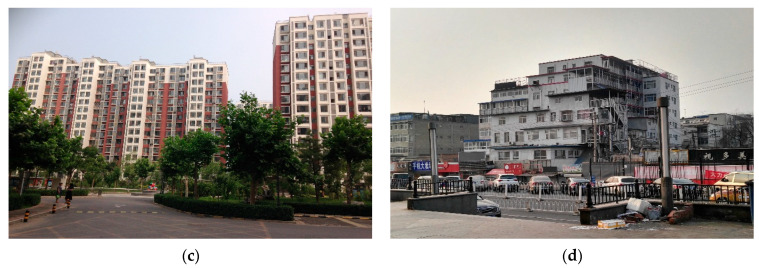
Housing tenure types accessible for migrants in the Beijing metropolis in China (**a**) commercial housing for self-owning or renting; (**b**) dormitory provided by employers in the industrial park; (**c**) public rented housing prepared for talented migrant workers; (**d**) “urban village” housing. Photos were taken by authors.

**Figure 2:**
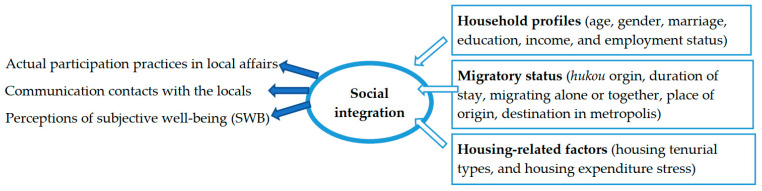
The three-tiered measurement of social integration (practical, relational, and perceptual), and its determinants (household, migratory, and housing-related). Originated and adapted from Ager and Strang’s conceptualization and Chen and Wang’s empirical studies in the Chinese megacity contexts [52,54].

The typical housing tenure opportunities available to the migrants in Beijing are composed of: (a) self-owned commercial housing; (b) dormitory-like housing provided by governments or employer units; (c) rental commercial housing; and (d) informal housing involving “urban village” housing, small property housing and other types of informalities [55,56]. Figure 1 displays the main housing types accessible to the migrants in Beijing, and Figure 2 demonstrates the conceptual framework that constructs and interprets the aging pre-1970 migrants’ integration into Beijing’s local society. As shown in Figure 2, we construct a three-tiered measurement of integration (practical, relational, and perceptual), and examine the main determinants of integration (from perspectives of household, migratory, and housing-related) to interpret the variance of aging migrants’ integration levels. The housing-related explanatory factors involve housing tenure types and housing expenditure stress. The definitions and measurements of dependent and explanatory factors will be elaborated in Section 3, based on the data availability of the 2017 MDMS.

Considering that the pre-1970 migrants straddle a wide range of life courses, we try to separate the whole pre-1970 migrants into two age cohort groups according to China’s retirement policy: (a) ≥60 years old (mostly retired), and (b) pre-1970 and <60 years old (mostly still working). They are facing quite different situations, and the housing impact on their respective social integration would be quite different, too.

We hypothesize that:

**Hypothesis** **1:** *The insecurities of housing tenure can lower the aging migrants’ integration, and home-owning can beef up their integration; and*

**Hypothesis** **2:** *Those in a paid employment status (<60 years old) vs. those in a retired status (≥60 years old) would indicate the differentiated integration processes faced by the aging migrants*.

## 3. Data and Method

### 3.1. Research Area and Data Source

The data is derived from the 2017 Migrant Dynamics Monitoring Survey (MDMS) collected and issued by the National Health Commission in China. This survey adopted a stratified three-stage probability proportion to size (PPS) random sampling method and involved the investigation questions on the migrants’ social integration in the destination city. We selected the migrant samples collected in the Beijing metropolis. In total valid 6999 samples in Beijing, we select 1267 samples of pre-1970 generation migrants (aged between 48 and 84 years old), who were born before 1970 and became the first-generation migrants in reformist China but getting aging now. The migrants had lived in Beijing for one month or more but did not get local *hukou* in Beijing.

We choose Beijing as the case study. First, Beijing is the most important destination city of the Chinese domestic migration in the north, accommodating migrants with different economic-cultural backgrounds in a diversity of housing types. Migrants’ housing choice in Beijing was repetitively investigated in previous studies [57,58], and the Beijing metropolis is deemed an ideal case to study the integration of diverse migrant populations including the aging migrants. Second, Beijing itself has a large elderly population, and it is believed that the inflow of younger migrants can slow down the process of population aging. Policy makers paid more attention to how to serve the aging local population and attract educated young migrants while ignoring the integration and health issues of the aging migrant populations. For instance, recent studies investigated the role of changing the urban environment in the everyday aging experiences in Beijing, and the surveys were conducted on the older people living at home in Beijing [59]. It was found that the growing housing inequality in post-reform urban China reflects how older people assess their built environment change in the Beijing metropolis, but such studies did not focus on the aging migrant populations. More research is needed to fill in this research gap.

**Figure 3:**
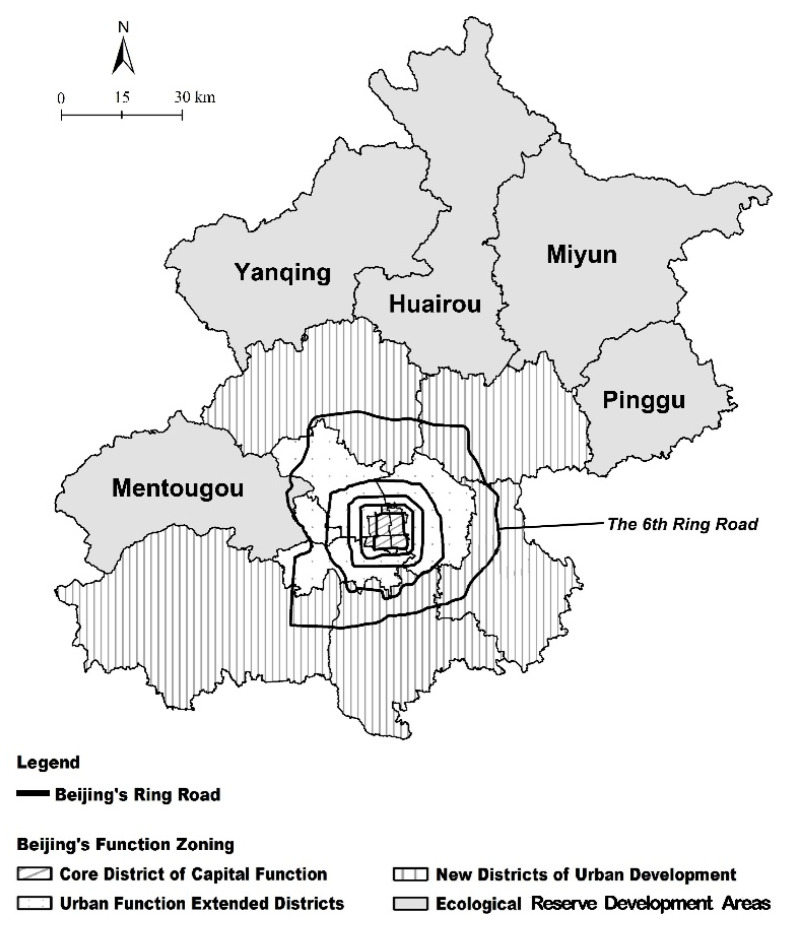
Map for the Beijing Municipality and its main functional zones.

The purpose of this study is to understand the role of different housing tenure types (i.e., self-owned housing; dormitory-like housing provided by governments or employers; rental housing; and informal housing, see Figure 1) in the integration of the aging migrants into the Chinese metropolises such as Beijing. The statistical analysis will be conducted in the next section. The self-owned commercial housing is the most “secure” housing type and a place more like a “home” for migrants. The surveyed aging migrants displayed a higher home-owing ratio (30.1%) than an average level in the total migrant samples (19.8%) in Beijing, due to their higher capacity to acquire an owned home after years of adaptation and success, or due to their kids’ home-owning status in Beijing. Interestingly, there is little variance between the proportion of aging migrants living in informal housing (29.80%) and the average level of total migrant populations (33.6%). It is thus indicated that the associations between housing inequality and stratification would be greater with the aging migrants than it is in the total migrant populations. Informal housing in Beijing is highly concentrated in Chaoyang, Haidian, and Fengtai districts in the Urban Function Extended Districts. The map for Beijing Municipality is shown in Figure 3. Additionally, the dormitory-like housing proportion of the aging migrants (14.7%) keeps the same as the total migrant population (12.6%), too. However, living in the dormitories often means sharing a room with other workers, and for this reason, dormitories would not be the first choice for many migrant households.

### 3.2. Variable Selection and Methodology

Table 1 defined the dependent variables and explanatory variables in this study. As elaborated earlier, we construct a three-tiered measurement of integration (practical, relational, and perceptual). The aging migrants’ social integration is defined as listed in Table 1. The relevant questions were listed in the questionnaire of the 2017 MDMS, which contains the specific “social integration” section.

First, from a practical dimension, we assign the value of 1 to the “local affair participation” variable, if the aging migrants had participated in the local affairs in Beijing (e.g., the labor union, volunteers’ association, supervising or offering advice for the management of their workplace and community, reporting to governments and giving policy making suggestions, online comments and discussions on the national affairs and social news, donating, blood donation, volunteer activities, and participating in the Party’s branch meetings and activities). Otherwise, we give the value of 0 to the 1st tier of the dependent variable.

Second, from a relational perspective, we assign the value of 1 to the 2nd tier of the dependent variable “communication with the locals as the most frequent daily contacts”, if the aging migrants have chosen the local Beijingese as the primary communication contacts instead of their own family- and clan-relationship or other populations. Otherwise, we give the value of 0 to this tier of the dependent variable.

Third, from a subjective and perceptual definition, we assign the value of 1 to the 3rd tier of the dependent variable “a full integration status perceived”, if the aging migrants give full marks in the questionnaire questions concerning migrants’ perceptions of subjective well-being (SWB). The five positive and inclusive settings of subjective well-being (SWB) are inquired about in the five questions of the 2017 MDMS: (a) I like the city I am living in now; (b) I pay attention to the change of this city; (c) I am willing to integrate into the local society and become a member; (d) I think the locals are willing to accept me as one of them, and (e) I think I am already a “local” resident. Each of the five questions is assigned 1 to 4 points to the four choices (i.e., complete disagreement, disagreement, basic agreement, and complete agreement, respectively). If the aging migrants gave a full score of 20 for the five questions, we consider them able to integrate fully into the local city and assign the value of 1 to this dependent variable. Otherwise, we give the value of 0 to the 3rd tier of the dependent variable.

Next, we examine the main determinants of integration and select three types of attributes including household profiles, migratory status, and housing-related factors to interpret the variance of the aging migrants’ integration level. Table 1 lists a total of 13 exploratory variables in the above three attribute groups. The focus would be put on the impact of the different housing tenure on the social integration of the aging migrants in Beijing. The housing tenure categories and related landscapes were mentioned earlier in Figure 1.

### 3.3. Regression Model Selection

This paper uses a binary logistic regression model to study the associations of social integration among the aging migrants with differential housing tenures. The calculation formula is listed as follows:

(1) $$\log\left[\frac{p}{1-p}\right]=a+b_{1}X_{1}+b_{2}X_{2}+b_{3}X_{3}+\text{…}+b_{k}X_{k}$$

where X_1_, X_2_, X_3_ … X_K_ are independent variables, *a* is a constant term, b_1_, b_2_, b_3_… b_K_ represent the regression coefficients of the respective explanatory variables.

The dependent variables are participation, communication contact, and subjective identity, as listed in Table 1. *P* is the probability of being willing to integrate, and 1-*P* is the probability of not planning to integrate.

Logistic regression is widely used when the dependent variables are categorical (e.g., No = 0, Yes = 1 as listed in Table 1). Regression results are reported in the results section.

## 4. Results

### 4.1. Descriptive Statistical Analysis

The descriptive statistics are summarized in Table 2. Most of the aging pre-1970 migrants were living in self-owned commercial housing (30.1%) and informal housing such as “urban villages” (29.8%). Such a tenurial divide often displays a large socioeconomic difference and a contrasting integration status.

The aging migrants living in owned housing have probably the family members (e.g., spouse, kids, son/daughter-in-law), who have already acquired Beijing *hukou*. Compared with the other three types of housing, these aging occupiers demonstrate the obvious advantages in all the practical, relational, and perceptual dimensions of integration, reportedly, 54.5% participate in the local affairs, 40.6% list communication with the locals as a prominent daily contact means, and 30.4% regarding themselves fully integrated into local society. This more inclusive status can be evidenced by the fact that 84.0% of these aging home-occupiers have got retired or are unemployed, and 78.5% of them have an urban origin (instead of a rural origin), as listed in Table 2.

A comparative study on the integration level is quite illuminating, between the aging migrants living in (a) dormitory-like housing provided by the governments or employers, (b) rental commercial housing, and (c) informal housing. It is believed that those in informal housing are more disadvantaged and segregated. However, the relational and perceptual measurements of integration (communication contacts index and SWB index in Table 2) in the informal housing are reported at the same level or even higher than that in the dormitory-like housing provided by the governments or employers. The values of SWB are also the same between those living in rental commercial housing and informal housing.

It is impressive that those living in the formal housing are congregated in the Urban Function Extended Districts in Beijing, while those in informal housing are present mostly in the New Districts of Urban Development in Beijing (see Table 2). A massive demolition of urban villages in Haidian, Fengtai, and Chaoyang Districts since 2009 can explain such a geographical disparity between the formal and informal housing in Beijing [60].

### 4.2. Regression on the Pre-1970 Migrants’ Integration Status

We have tested how to partition the whole pre-1970 migrants into two cohorts (majority still working vs. majority being retired), who are at two very distinct life stages. In sum, 48–59 years old are more likely in a still working status (labor force ratio as high as 78.2%), and this situation is consistent with China’s retirement policy that men retire at 60 and women retire at 55. We thus select 60 years old as a threshold to stratify the whole pre-1970 migrants into (a) 48–59 years old cohort and (b) ≥60 years old for further comparative studies. The majority (78.2%) of the 48–59 years old age cohort are still working. According to the 2017 MDMS, it is reported that among the still working 48–59 years old age cohort, 59.7% of them are factory workers in the secondary industry assuming hard physical labor jobs. 8.3% of them came to Beijing to continue their agricultural activities. Only 7.4% of them are white-collars with more decent jobs in the governments, enterprises, and institutions. Few (0.6%) are engaged in the service work. The remaining 2.2% of them engage in flexible work.

Table 3 summarizes the main findings from the regression analysis. We will elaborate on the analysis results in the three models on the three-tiered measurement of social integration (practical, relational, and perceptual), respectively.

#### 4.2.1. Explaining the Practical Dimension of Aging Migrants’ Integration

Participation in local affairs (a binary variable: yes or no) is an indicator of aging migrants’ integration measured by their actual participation practices (regardless of a small group-like community/neighborhood or the mass society of a city).

First, in the whole pre-1970 migrant population, the regression results in Table 3 display that a younger age (*p* < 0.05, OR = 0.976), higher educational level (significant, OR < 0.7), an employed status (*p* < 0.1, OR = 1.390), a longer stay in Beijing (*p* < 0.001, OR = 1.043) would be the significant indicators for those more active in local participation. Among all the indicators, human capital is the strongest to predict the aging migrants’ probability of participation in local affairs. Well-educated aging migrants are more active in local affairs participation. The probability of local affairs participation in poorly educated populations (primary and below) is merely 0.251 times (*p* < 0.001), compared with those with a college degree and higher. It is thus proved that a lower educational background presents a significantly negative correlation to participation. Housing tenure type is proved the second strongest indicator to explain the variance of aging migrants’ local affairs participation. Homeowners are more likely to participate in the local affairs in Beijing than those living in informal housing (*p* < 0.001, OR = 2.641).

Second, the regression analysis on the two age cohorts (48–59 years old and ≥60 years old) has reported the similar findings that younger age (*p* < 0.001, OR = 0.908; *p* < 0.05, OR = 0.942), higher educational level (significant, OR < 0.6), and a longer stay in Beijing (significant, OR = 1.041, OR = 1.061) are the significant indicators for those more active in local participation. The employed status is an insignificant factor when modeling the two age cohorts. There has been a decisive divide in employment status between the two age cohorts, and for this reason, the within-group variance of employment status in each age cohort becomes minor. Moreover, the housing tenure factor displays different associations with the participation in local affairs in Beijing. For instance, for the 48–59 age cohorts who are still working, living in the dormitory-like housing indicates a significantly higher odds of local affairs participation than living in the owned homes.

#### 4.2.2. Explaining the Relational Dimension of Aging Migrants’ Integration

From a relational perspective, we use the “whether communication with the locals as the most frequent daily contacts” (the binary variable: yes or no) as the second dependent variable in this study. Daily communication contact is an important means for the aging migrants to integrate into mainstream society.

First, in the whole pre-1970 migrant population, regression results in Table 3 show that the female aging migrants (*p* < 0.1, OR = 0.759), those better educated (*p* < 0.05, OR = 0.490), an urban *hukou* origin (*p* < 0.05, OR = 0.599), and the longer stay in Beijing (*p* < 0.001, OR = 1.040) are more likely to list the local *Beijingese* as the primary communication contacts (instead of their own family- and clan-relationship or other populations). It is also found that geographical and locational-related factors matter. It is reported in Table 3 that the aging migrants whose place of origin shares similarities (in terms of customs, cultures, and languages) with Beijing are more easily inclusive in this contact dimension. Those from the South-Western Region in China (*p* < 0.05, OR = 0.400) face more barriers to integration. Beijing as a destination city is more inclusive for those migrating from vast North China. Moreover, those choosing a residential location in Beijing’s more peripheral Ecological Reserve Development Areas (significant, OR < 0.5) are more probably interactive with the local *Beijingese*. Aging migrants living in the urban centers are more easily excluded from the local people’s network. However, these peripheral areas are more inclusive, as native people in peripheries hold an “outsider identity” in the capital-city culture which is urban-center-dominated. In addition, Ecological Reserve Development Areas are located in the nature-human interface, creating a better contact and recreation atmosphere for the aging migrants. More research can be conducted to explore the inter-variance of integration between different locations within a metropolis.

The impact of housing tenure type on the aging migrants’ daily communication contacts is significant (*p* < 0.05, OR = 1.909), but not as prominent as those when modeling the associations with local affairs participation. Compared with those in informal housing, homeowners are significantly more interactive and communicative with the local *Beijingese* (*p* < 0.05, OR = 1.909). However, such a contact difference is insignificant between those living in the dormitory-like housing (provided by governments or employer units), rental commercial housing, and informal housing.

Second, the regression analysis on the two age cohorts (48–59 years old and ≥60 years old) has reported that the gender is insignificant. The education level and *hukou* origin are insignificant for older age cohorts (≥60 years old), but remain significant for younger still working age cohorts (48–59 years old); higher educational level (significant, OR < 0.5), and urban *hukou* origin (*p* < 0.05, OR = 0.588) are the significant indicators for still working groups’ daily communication contacts. The longer stays in Beijing (significant, OR = 1.044, OR = 1.037) are a significant indicator for both age cohorts’ daily communication contacts. The similarity or disparity between the place of origin and Beijing also plays a significant role in the older or younger age cohorts’ local communications. A similar inter-variance of integration between different locations within a metropolis is reported, too, in the modeling of the two age cohorts (48–59 years old and ≥60 years old). However, housing is found to have different associations with the younger or older cohorts’ daily communication contacts. For instance, when younger still working age cohorts (48–59 years old) are living in the owned homes, they have significantly higher odds of local communications than those living in informal housing. However, when older age cohorts (≥60 years old) are living in the dormitory-like housing, they would report the highest odds of local communications. The complicated two- or three-generation co-living status can explain such a difference for the retired migrant populations.

#### 4.2.3. Explaining the Perceptual Dimension of Aging Migrants’ Integration

Subjective well-being (SWB) is a widely used integration indicator in previous studies. Interestingly, human capital did not report significant associations of educational levels with the aging migrants’ integration.

First, in the whole pre-1970 migrant population, the subjective identity of retired or unemployed aging migrants is significantly stronger than that of their still working peers (*p* < 0.05, OR = 0.574)—that is, the probability of a full integration status perceived (SWB) among bread earners is only 57.4% of that for the retired or unemployed aging migrants. Age discrimination against job seekers has a trend of convergence towards 45 years old [9]. Disadvantaged aging migrants face more difficulties adapting to the age limit in the employment markets. However, those who have retired may have more pride than the migrant workers, as they dwell with kids who have probably already acquired Beijing *hukou*. In addition, a longer stay in Beijing (*p* < 0.001, OR = 1.044) predicts a higher probability of full integration status perceived (SWB) by the aging migrants. Those living in the “Urban Function Extended Districts” report the lowest probability (61.4%) to perceive a fully integrated status (SWB), compared with those in the Ecological Reserve Development Areas (*p* < 0.1, OR = 0.614). A rampant migrant enclave demolition in the Urban Function Extended Districts can explain this result [60].

The homeowners report the highest probability of perceiving a fully integrated status (SWB), compared with those living in informal housing (*p* < 0.05, OR = 1.923). Interestingly, those dwelling in the dormitory-like housing (from governments or employers) report a lower ratio of perceiving a fully integrated status (SWB) than those living in informal housing, despite that the regression coefficient is insignificant. More case studies are needed to explore the specific reasons behind it.

Second, the regression analysis on the two age cohorts (48–59 years old and ≥60 years old) has reported that a longer stay in Beijing can predict a higher probability of full integration status perceived (SWB) in both the two age cohorts. The employment status is still significant for older age cohorts (≥60 years old), but insignificant for younger still working age cohorts (48–59 years old)—that is, the odds of the full integration status perceived (SWB) among the bread earners in the older age cohorts (≥60 years old) is merely 15.5% of that for the retired or unemployed peers. It is also reported that the education level is significant for younger still working age cohorts (48–59 years old); a higher educational level can predict a higher odds of full integration status perceived (SWB) for still working groups. Interestingly, housing tenure is insignificant for the older age cohorts’ (≥60 years old) full integration status perceived (SWB), but home-owning plays a significant role to predict the higher odds of the full integration status perceived (SWB) among the younger still working age cohorts (48–59 years old). Among the older age cohorts who have retried, homeowners in Beijing are probably their sons or daughters. The household-level tenure- and *hukou* structure within the two- and three-generation relationships can shed light on this nuance.

## 5. Discussion

### 5.1. Reinterpreting Social Integration in the Chinese Urbanizing Regime

One of the most classic theories concerning social integration is assimilationism, which was originally put forward by the Chicago School during the prosperous Western industrialization and immigrant flow-in periods before World War II [61]. Social integration has been defined as a process of fusion, in which immigrants and locals adopt the memories, sentiments, and attitudes of one another, and through such incorporation form a common cultural life. Interestingly, the Chicago School drew on Emile Durkheim and Herbert Spencer’s thoughts to understand the complex processes of socio-spatial differentiation that involve both social fragmentation and social integration (namely the secondary institutions performing the crucial social integration functions) and at the same time can achieve relative stability and equilibrium.

In the post-World War II studies in the OECD countries, social integration refers to a person’s sense of belonging or attachment. As defined by Keyes (1998: 122), integration is “the extent to which people feel they have something in common with others who constitute their social reality, as well as the degree to which they feel that they belong to their communities and society” [62]. As revealed by Fischer in his review of the social integration literature, the older adults, homeowners, the better educated, the wealthy, women, whites, longtime residents, and people with children were found to be more socially integrated than their younger, renting, less-educated, poorer, male, nonwhite, short-term residents, and childless counterparts [63]. In addition to those social status characteristics, urbanism has close associations with social interaction, too, though no clear consensus exists. For instance, Fischer suggested that people in urban centers are just as socially integrated and satisfied with their lives as suburban residents if analysis controls for socioeconomic and demographic factors [63]. Community and investments in it have associations with social integration, too. Long-term residents and homeowners are found more likely to participate in local events and have friends, and for this reason, home ownership and length of residence are treated as indicators of social ties and integration [64,65,66].

However, new waves of immigration in the post-World War II had transformed American cities into spaces of ethnic diversity and reproduced a highly differentiated social landscape in contemporary metropolises such as Los Angeles. One of the central interests of urban studies has been the quest to understand the extent of social isolation or social segregation, which is the opposite of social integration. The Los Angeles School drew from Marxist, poststructuralist, and postmodern theories to interpret the city as a highly differentiated, conflictual, and divided space. Social isolation or social segregation represents a breakdown of social integration that otherwise can provide social support or a sense of belonging. The social and spatial dimensions of integration (or lack of it) are considered interchangeable in the emerging “divided cities” in the USA, but Musterd, Ostendorf, and Galster pointed out it is still unclear whether more social contact or neighborhood mixing between different people does indeed result in a higher level of integration [50,51,67]. The UK attracted most immigrants from former colonies in the post-World War II period but triggered some serious conflicts, too. The focus is on how to establish social unity and welfare equality in a culturally diverse society. Adachi defined social integration as a situation in which social unity co-exists with cultural diversity, and people with different backgrounds can share the same concept of society [68].

Under the profound impact of the *hukou* system, the definition and demonstration of social integration in China are different from that in the Western contexts. For instance, the existing studies on the massive internal migration in China paid much attention to the integration, permanent settlement, and *hukou* transfer initiatives. However, little attention is given to the integration of the pre-1970 first-generation migrants, who were born before the year 1970 and getting old in the destination cities. The pre-1970 migrants have a wide range of age cohorts in urban China: (a) those over 45 years old and still working but facing the age discrimination challenges in employment markets; and (b) de facto elderly migrants who are already retired but also facing the difficulties adapting to living environments in the destination city, because they assume the domestic and child-care work for their sons or daughters but have few chances for out-of-home leisure or local communication contact.

In this study, we have adopted and improved Ager and Strang’s conceptualization of social integration, as well as Chen and Wang’s migrant integration analysis framework in the Chinese megacity contexts [52,54], and then re-defined aging migrants’ social integration in an innovated triple dimension (i.e., participation practices, communication contacts, and subjective perceptions). We use the social integration data from Beijing’s aging migrant samples, which were collected from the 2017 Migrant Dynamics Monitoring Survey (MDMS), to measure and explain the varied integration levels among a total of 1267 aging migrant samples. The three dimensions of social integration in the Chinese urbanizing context can fill the practical, relational, and perceptual/psychological aspects in conventional social integration studies.

### 5.2. Main Findings

First, the housing tenure security has proven to be a strong indicator to explain the variance of aging migrants’ local affairs participation (Hypothesis 1). When explaining the local affairs participation of all the pre-1970 migrants, the practical-dimensional integration stays almost the same when living in a dormitory-like housing (provided by local governments or employer units) or a rental commercial housing acquired from markets (OR = 1.607, OR = 1.645, respectively). It thus indicates that the formality/informality is an important indicator of aging migrants’ local affairs participation. However, the variance of suppliers (governments, employers, markets) is not a convincing indicator of integration levels. Home occupation matters in integration. The associations between tenure and local affairs participations vary with the different age cohorts. For instance, in all pre-1970 migrant populations, homeowners are more likely to participate in local affairs than those living in informal housing. A similar conclusion stands when it refers to the older age cohorts who are above 60 years old and mostly retired. Interestingly, the analysis did not detect a significant variance of tenure-participation nexus between home-owning and informal housing dwelling for the 48–59 age cohorts who are still working. For the 48–59 age cohorts, living in the dormitory-like housing indicates the significantly higher odds of local affairs participation than living in the owned homes. A relatively low proportion of the 48–59 age cohorts in well-paid occupations can explain the homeowning-participation paradox in the still working groups.

Second, the analysis results also show that the life cycle matters (Hypothesis 2), and the analysis reveals the employment-income paradox in explaining aging migrants’ social integration in Beijing. In the whole pre-1970 migrants in Beijing, a higher income level predicts a lower probability of local affairs participation (*p* < 0.05, OR = 0.610), which was in contradiction with previous research findings. The bread earners report a higher ratio for local affairs participation (*p* < 0.1, OR = 1.390) than those retired or unemployed, while the income level has an opposite association with local affairs participation (*p* < 0.05, OR = 0.610). Such an employment-income paradox is interpretative when there exists a large group of retired migrants, living with their sons or daughters and caring for grandchildren in the destination city. Their aggregated household income is reported high, but “granny as a nanny” has little time for out-of-home leisure and local communication contacts, when assuming domestic and child-care work for sons or daughters. This reported high-income status is accompanied by the specific segregation status, as “granny as a nanny” has left their familiar environment and social networks in their hometown while floating in the destination city. This demonstrates that higher economic achievement is NOT equivalent to a higher social integration status for the aging migrants in a great metropolis such as Beijing.

Third, we can also detect a complex interplay between housing tenure heterogeneity and life cycle heterogeneity (Hypothesis 1 × Hypothesis 2). For instance, for the whole pre-1970 migrant population, the homeowners are significantly more interactive and communicative with the local *Beijingese* (*p* < 0.05, OR = 1.909) than those in informal housing. However, housing is found to have different associations with the different cohorts’ communication contacts. Homeownership is still a significant indicator for the younger still working age cohorts’ (48–59 years old) local communication contacts. Yet when it comes to the older age cohorts (≥60 years old), those living in the dormitory-like housing have reported the highest odds of the local communications. Further research is needed to explore the two- or three-generation co-living structure. More detailed household-level data analysis can explain this nuance. It is also found that housing tenure is insignificant for the older age cohorts’ (≥60 years old) full integration status perceived (SWB), but home-owning plays a significant role to predict higher odds of the full integration status perceived (SWB) among the younger still working age cohorts (48–59 years old). Among the older age cohorts who have retried, homeowners in Beijing are probably their sons or daughters. The household-level tenure- and *hukou* structure within the two- and three-generation relationships can shed light on this nuance.

## 6. Conclusions

The data analysis findings are illuminating to guide follow-up studies. First, housing tenure matters. The securities of migrants’ housing tenure (i.e., migrants as home-occupiers in the destination cities) can significantly beef up their integration, while the insecurities of housing tenure (e.g., informal housing) can lower their integration. Housing tenure entitlement is therefore conducive to beefing up the aging migrants’ integration. This research result can fulfill the findings from previous studies. For instance, Lin et al. divided housing into different types, and then put them into three models to measure the migrants’ urban attachment [69]. Yang et al. also used different types of urban villages in Shenzhen as an independent variable to analyze their impact on the migrants’ social integration [70]. Our empirical study contributed to another case study on Beijing’s pre-1970 migrants, and further supported the conclusion that the housing tenure security and entitlement are conducive to the aging migrants’ social integration process.

Second, different indicators exert a differentiated impact on the three-tiered measurement of social integration (practical, relational, and perceptual). For instance, the associations of human capital with integration are significant in the practical and relational dimensions, but insignificant in the perceptual dimension. The contrast between the paid employment status vs. those in a retired status (see the indicator of “employment status”) has indeed reported the differentiated impact on integration processes, and this differentiation is significant in the practical and perceptual dimensions, but insignificant in a relational dimension. This research also pointed out an employment-income paradox, which shows that higher economic achievement is NOT equivalent to a higher social integration status for the aging migrants. Their aggregated household income is reported high, but the “granny as a nanny” faces segregation problems in terms of local affair participation. More detailed surveys on representative migrant households would be helpful to figure out the specific mechanism behind such an employment-income paradox on integration. It is also reported that the still working migrants have less satisfaction or pride than their retired peers in the perceptual dimension of integration. The retired groups may dwell with their kids who have probably already acquired the Beijing *hukou*, and probably, for this reason, a retirement status indicates a higher probability of full integration status perceived (SWB). The family data of the 2017 MDMS shows that: (a) among all the pre-1970 samples, 224 households are co-living between the granny/grandpa and the grandson/granddaughter (approaching 20% in all the targeted pre-1970 samples); and (b) interestingly, among them, 115 grandsons/granddaughters hold the local Beijing *hukou*. Such a unified three-generation family structure can support the saying of “granny as a nanny”, which is a popular “*laopiao*” phenomenon in Beijing. Further research is needed to probe into the complicated *hukou* arrangement within a family and its impact on the aging people’s social integration.

Finally, is informal housing a sign of segregation? The answer is uncertain and varies with specific case studies. As reported in Table 2 concerning with communication contacts index and SWB index, those dwelling in informal housing have reported the same or even higher level of integration, compared with those living in the dormitory-like housing provided by the local governments or employers. The regression results also report that the contact variance is insignificant between those living in informal housing, dormitory-like housing, and rental commercial housing. It is also reported that those dwelling in the dormitory-like housing have a lower ratio of perceiving a fully integrated status (SWB) than those living in informal housing, despite that its regression coefficient is insignificant. Is it reasonable to justify the dispossession by stigmatizing informal housing as a segregated world [71]? More empirical studies are needed to reveal the relational and functional nature of the so-called “informal” property rights in transitional China.

This research contributed another empirical study on Beijing’s aging migrants to social integration studies. First, the aging of the global population is the most important medical and social demographic problem worldwide. The World Health Organization (WHO) has defined healthy aging as a process of maintaining the functional ability to enable well-being in older age. This article focuses on social integration as crucial to measuring the healthy aging of migrants, filling for the practical, relational, and perceptual/psychological aspects (see Figure 2) of healthy aging studies. The second contribution is the findings on the housing tenure impact on the social integration of the pre-1970 first-generation migrants in reformist China. Hypothesis 1 on housing tenure heterogeneity is proved in our study. However, it is found that those living in the informal housing have a higher probability of perceiving a fully integrated status (subjective well-being, SWB) than those living in the formalized dormitory-like housing. These findings shed light on the housing formalization policies in megacities in China: the physical demolition of migrant enclaves would also cut down their active social bonding and positive subjective well-being, which deserves more attention in policy making. More comparative studies on the social impact of informal migrant enclaves in the fast-urbanizing countries (i.e., China, India, and Latin America) can shed light on the global debates on “ageism and urbanization” [72]. For instance, is the destination city age-friendly, and how can we promote the urbanization pattern through the lens of old age? Third, our research reveals the geographies of aging among migrants, and different situations between younger (48–59 years old) and older (≥60 years old) of the pre-1970 cohorts. Hypothesis 2 on the life cycle heterogeneity is proved, too. This nuance of aging migrants deserves more attention in further studies.

As elaborated above, the strengths of this research are manifested: (a) a more comprehensive definition of aging migrants’ social integration, filling for the practical, relational, and perceptual/psychological aspects (see Figure 2) of healthy aging studies; (b) critical thinking on the role of informal housing and migrant enclaves in the migrants’ integration that can shed more light on the global debates on “ageism and urbanization”; and (c) the nuance of aging migrants in terms of “housing tenure heterogeneity” (Hypothesis 1) and “life cycle heterogeneity” (Hypothesis 2) in their social integration processes. The weakness of this study is that we have started to pay attention to the complicated life cycle and the family-based *hukou* package that have exerted profound impact on the aging migrants’ integration into Beijing. However, we did not investigate and reveal their mechanism at a more detailed household scale. Further research can depict the specific impact of life cycle and household-level strategies (such as the *hukou* package within a family and three-generation relations) on the aging migrants’ social integration. Policy makers should take some conservative interventions in the informal housing management, including providing more age-friendly environments, facilities, and services in the migrant enclaves. The age-friendly *hukou* reforms are needed, too, to entitle more benefits for the two- or three-generation migrant households.

## Figures and Tables

**Table 1:** Indicators.

	Indicators	Descriptions	Mean	Standard Deviation
***Dependent variables***				
	1 Local affairs participation	No = 0, Yes = 1	0.44	0.49
	2 Communication with the locals as the most frequent daily contact	No = 0, Yes = 1	0.26	0.44
	3 A full integration status perceived (subjective well-being)	No = 0, Yes = 1	0.19	0.39
***Explanatory variables***				
Household profiles	Age (years old)	Continuous variable	57.74	8.25
	Gender	Male = 1, Female = 2	1.43	0.50
	Marital status	Married = 1, Unmarried = 2	1.10	0.30
	Educational attainment	Primary and below = 1, Junior secondary = 2, Senior/technical secondary = 3, College and above = 4	2.22	0.96
	Household monthly income (logged, unit: yuan)	Continuous variable	3.85	0.33
	Employment status	Employed = 1, Unemployed or retired = 2	1.41	0.49
Migratory status	Hukou origin	Rural hukou = 1, Urban hukou = 2	1.42	0.49
	Duration of stay in Beijing (years)	Continuous variable	9.73	7.73
	Migrate alone or together	Yes (migrate alone) = 1, No (migrate together with family) = 2	1.96	0.21
	Place of origin	Eastern Region = 1, North Region = 2, Central Region = 3, South-Western Region = 4, North-Western Region = 5, North-Eastern Region = 6	3.18	2.22
	Destination in Beijing metropolis	Core District of Capital Function = 1, Urban Function Extended Districts = 2, New Districts of Urban Development = 3, Ecological Reserve Development Areas = 4	2.24	0.77
Housing-related factors	Housing tenure types	Self-owned commercial housing = 1, Dormitory-like housing provided by governments or employer units = 2, Rental commercial housing = 3, Informal housing = 4	2.55	1.20
	Housing stress (housing expenditure-to-income ratio)	Continuous variable	0.14	0.39

Note: 1. The rare samples from the South-Eastern Region (N = 4) are detected as outliers and are deleted for this reason. 2. In the Beijing metropolis, (a) Core District of Capital Function (Inner Cities) includes Dongcheng and Xicheng Districts; (b) Urban Function Extended Districts (Inner Suburbs) include Chaoyang, Fengtai, Shijingshan, and Haidian Districts; (c) New Districts of Urban Development (Outer Suburbs) includes Fangshan, Tongzhou, Shunyi, Changping and Daxing Districts; and (d) Ecological Reserve Development Areas (Mountainous Areas) includes Mentougou, Huairou, Pinggu, Miyun and Yanqing Districts. Data source: MDMS, 2017.

**Table 2:** Descriptive statistics of the aging pre-1970 migrants across the different housing tenure types in Beijing.

		Self-Owned Commercial Housing		Dormitory-Like Housing Provided by Governments or Employer Units		Rental Commercial Housing		Informal Housing		Total	
Sampling		382	30.1%	186	14.7%	321	25.3%	378	29.8%	1267	100%
***Dependent variables***											
1 Local affairs participation	Yes	208	54.5%	88	47.3%	145	45.2%	122	32.3%	563	44.4%
	No	174	45.5%	98	52.7%	176	54.8%	256	67.7%	704	55.6%
2 Communication with locals as the most frequent daily contact	Yes	155	40.6%	34	18.3%	67	20.9%	70	18.5%	326	25.7%
	No	227	59.4%	152	81.7%	254	79.1%	308	81.5%	941	74.3%
3 Full integration perceived (SWB)	Yes	116	30.4%	20	10.8%	49	15.3%	57	15.1%	242	19.1%
	No	266	69.6%	166	89.2%	272	84.7%	321	84.9%	1025	80.9%
***Explanatory variables***											
**Household profiles**											
Gender	Male	181	47.4%	124	66.7%	179	55.8%	237	62.7%	721	56.9%
	Female	201	52.6%	62	33.3%	142	44.2%	141	37.3%	546	43.1%
Marital Status	Single	39	10.2%	27	14.5%	23	7.2%	36	9.5%	125	9.9%
	Married	343	89.8%	159	85.5%	298	92.8%	342	90.5%	1142	90.1%
Education	Primary and below	54	14.1%	51	27.4%	85	26.5%	132	34.9%	322	25.4%
	Junior secondary	103	27.0%	82	44.1%	139	43.3%	166	43.9%	490	38.7%
	Senior/technical secondary	127	33.2%	48	25.8%	67	20.9%	65	17.2%	307	24.2%
	College and above	98	25.7%	5	2.7%	30	9.3%	15	4.0%	148	11.7%
Employment status	Employed	61	16.0%	164	88.2%	237	73.8%	283	74.9%	745	58.8%
	Unemployed or retired	321	84.0%	22	11.8%	84	26.2%	95	25.1%	522	41.2%
**Migratory status**											
*Hukou* origin	Rural *hukou*	82	21.5%	144	77.4%	217	67.6%	294	77.8%	737	58.2%
	Urban *hukou*	300	78.5%	42	22.6%	104	32.4%	84	22.2%	530	41.8%
Migrate alone or not	Migrate alone	10	2.6%	19	10.2%	13	4.0%	15	4.0%	57	4.5%
	Migrate together with family	372	97.4%	167	89.8%	308	96.0%	363	96.0%	1210	95.5%
Place of origin	Eastern Region	77	20.2%	41	22.0%	80	24.9%	85	22.5%	283	22.3%
	North Region	121	31.7%	66	35.5%	84	26.2%	120	31.7%	391	30.9%
	Central Region	53	13.9%	36	19.4%	82	25.5%	73	19.3%	244	19.3%
	South-Western Region	19	5.0%	10	5.4%	23	7.2%	29	7.7%	81	6.4%
	North-Western Region	26	6.8%	8	4.3%	13	4.0%	4	1.1%	51	4.0%
	North-Eastern Region	86	22.4%	25	13.4%	39	12.2%	67	17.7%	217	17.1%
Destination in Beijing metropolis	Core District of Capital Function	38	9.9%	34	18.3%	58	18.1%	34	9.0%	164	12.9%
	Urban Function Extended Districts	256	67.0%	112	60.2%	220	68.5%	143	37.8%	731	57.7%
	New Districts of Urban Development	57	14.9%	29	15.6%	28	8.7%	166	43.9%	280	22.1%
	Ecological Reserve Development Areas	31	8.1%	11	5.9%	15	4.7%	35	9.3%	92	7.3%

Note: The rare samples from the South-Eastern Region (N = 4) are detected as outliers and are deleted for this reason. Data source: MDMS, 2017.

**Table 3:** Logistic regress on the pre-1970 migrants’ integration: (a) all pre-1970; (b) ≥ 60 years old; and (c) < 60 years old & pre-1970.

	All Pre-1970 Migrants in Beijing						≥60 Years Old						<60 Years Old & Pre-1970					
	*1. Local Affairs Participation*		*2. Communication with Locals as the Most Frequent Contacts*		*3. Full Integration Perceived (SWB)*		*1. Local Affairs Participation*		*2. Communication with Locals as the Most Frequent Contacts*		*3. Full Integration Perceived (SWB)*		*1. Local Affairs Participation*		*2. Communication with Locals as the Most Frequent Contacts*		*3. Full Integration Perceived (SWB)*	
Predictors	*Β*	*Exp(β)*	*Β*	*Exp(β)*	*Β*	*Exp(β)*	*Β*	*Exp(β)*	*Β*	*Exp(β)*	*Β*	*Exp(β)*	*Β*	*Exp(β)*	*Β*	*Exp(β)*	*Β*	*Exp(β)*
**Household profile**																		
Age (years old)	−0.024 **	0.976	−0.001	0.999	0.008	1.008	−0.060 **	0.942	−0.018	0.982	0.029	1.030	−0.096 ***	0.908	−0.081 **	0.923	0.042	1.043
Gender (ref = female)	0.016	1.016	−0.276 *	0.759	−0.048	0.953	0.051	1.052	−0.308	0.735	−0.511 **	0.600	0.101	1.106	−0.290	0.748	0.173	1.189
Marriage (ref = unmarried)	−0.276	0.759	−0.035	0.966	0.092	1.096	−0.303	0.739	0.254	1.277	0.731	2.078	−0.398	0.672	−0.821 *	0.440	−0.667	0.513
Education level (ref = college and higher)																		
Primary and below	−1.382 ***	0.251	−0.714 **	0.490	−0.338	0.713	−1.441 ***	0.237	−0.654	0.524	0.111	1.117	−1.089 **	0.337	−0.995 **	0.370	−0.978 *	0.376
Junior secondary	−1.002 ***	0.367	−0.326	0.722	−0.218	0.804	−1.182***	0.307	0.188	1.207	0.113	1.119	−0.649 *	0.523	−0.770 *	0.463	−0.868 *	0.420
Senior/technical secondary	−0.42 *	0.657	−0.172	0.842	0.300	1.349	−0.691**	0.501	−0.096	0.908	0.368	1.445	0.102	1.107	−0.224	0.799	−0.137	0.872
Logged familial income (annual, unit: yuan)	−0.494 **	0.610	−0.190	0.827	−0.228	0.796	−0.653 **	0.520	−0.246	0.782	0.100	1.105	−0.084	0.919	0.095	1.100	−0.753 *	0.471
Employment status (ref = retired/unemployed)	0.329 *	1.390	−0.228	0.796	−0.555 **	0.574	0.227	1.255	0.073	1.076	−1.864 **	0.155	−0.087	0.917	−0.054	0.947	0.123	1.131
**Migratory status**																		
*Hukou* origin (ref = urban *hukou*)	−0.227	0.797	−0.512 **	0.599	0.118	1.125	−0.265	0.767	−0.476	0.621	0.012	1.012	−0.212	0.809	−0.523**	0.588	0.324	1.383
Duration of stay in Beijing (years)	0.042 ***	1.043	0.039 ***	1.040	0.043 ***	1.044	0.059 **	1.061	0.037 **	1.037	0.056 **	1.058	0.040 ***	1.041	0.043 ***	1.044	0.036 **	1.037
Migrate alone or together (ref = with family)	0.131	1.140	−0.361	0.697	0.383	1.467	0.390	1.477	−0.751	0.472	1.113	3.044	0.045	1.046	−0.699	0.497	−0.565	0.568
Place of origin (ref = North−Eastern China)																		
Eastern Region	−0.079	0.924	−0.066	0.936	−0.165	0.848	−0.316	0.729	−0.042	0.959	−0.679 *	0.507	0.101	1.106	0.058	1.060	0.147	1.158
North China	−0.013	0.987	0.215	1.240	−0.251	0.778	−0.039	0.962	0.038	1.038	−0.377	0.686	−0.021	0.979	0.375	1.455	−0.201	0.818
Central China	−0.001	0.999	0.002	0.995	−0.040	0.961	−0.311	0.733	−0.066	0.936	−0.063	0.939	0.129	1.137	0.183	1.210	0.150	1.162
South−Western Region	−0.130	0.874	−0.917 **	0.400	−0.051	0.951	−0.502	0.605	−0.150	0.860	−0.821	0.440	−0.026	0.974	−1.751 **	0.174	0.389	1.475
North−Western Region	−0.032	0.969	0.660 *	1.936	0.523	1.687	0.201	1.222	0.903 *	2.468	0.740	2.095	−0.241	0.786	−0.020	0.981	−0.056	0.946
Destination in Beijing metropolis (ref = Ecological Reserve Development Areas)																		
Core District of Capital Function	0.328	1.388	−0.891 **	0.410	−0.377	0.686	0.349	1.417	−2.109 ***	0.121	0.163	1.177	0.431	1.539	−0.520	0.595	−0.615	0.541
Urban Function Extended Districts	0.406	1.501	−1.112 ***	0.329	−0.487 *	0.614	0.796 *	2.216	−2.320 ***	0.098	−0.078	0.925	0.238	1.268	−0.659 *	0.517	−0.434	0.648
New Districts of Urban Development	0.304	1.355	−0.709 **	0.492	0.106	1.112	0.792	2.208	−1.844 **	0.158	0.895	2.448	0.079	1.082	−0.248	0.780	−0.043	0.958
**Housing−related factors**																		
Housing tenure types (ref = Informal housing)																		
Self−owned commercial housing	0.971 ***	2.641	0.647 **	1.909	0.654 **	1.923	1.204 **	2.784	0.504	1.656	0.393	1.482	0.387	1.472	0.574 *	1.776	1.056 **	2.876
Dormitory−like from gov or employer units	0.475 **	1.607	0.142	1.153	−0.191	0.826	−0.002	0.998	0.797 *	2.219	0.146	1.158	0.697 **	2.008	−0.175	0.839	−0.428	0.652
Rental commercial housing	0.498 **	1.645	0.117	1.125	0.126	1.134	0.477	1.611	−0.145	0.865	0.083	1.086	0.401 *	1.493	0.121	1.128	0.183	1.201
Housing stress (expenditure−to−income ratio)	−0.214	0.807	0.212	1.237	0.219	1.244	−0.373	0.689	0.148	1.159	0.401	1.493	0.331	1.392	0.398	1.490	−0.479	0.619
**Constant**	2.857 **	17.415	0.800	2.226	−1.154	0.316	5.856 **	349.257	3.059	22.095	−4.642 **	0.010	5.088 **	162.008	4.089 *	59.675	−0.409	0.664
N	1267		1259		1267		495		495		495		772		772		772	
***df***	23		23		23		23		23		23		23		23		23	
*λ^2^*	152.633		141.969		95.174		83.028		65.398		68.257		96.555		92.361		45.571	
−2 Log−Likelihood	1577.344		1285.410		1131.423		590.522		564.252		476.144		959.008		670.304		621.15	
Nagelkerke *R^2^*	0.153		0.157		0.117		0.209		0.173		0.194		0.158		0.180		0.099	
Percent correctly classified	66.2%		76.2%		81.7%		67.2%		71.3%		75.4%		67.4%		81.1%		84.7%	

Note: Significant at * 0.1; ** 0.05; *** 0.001 level. The rare samples from the South-Eastern Region (N = 4) are detected as outliers and are deleted for this reason. Data source: MDMS, 2017.

## Data Availability

Data use of the 2017 Migrant Dynamics Monitoring Survey (MDMS) is supported and approved by the Migrant Population Service Center, the National Health Commission of China. https://www.chinaldrk.org.cn/wjw/#/home (accessed on 11 April 2022).
